# Synergism of AZD6738, an ATR Inhibitor, in Combination with Belotecan, a Camptothecin Analogue, in Chemotherapy-Resistant Ovarian Cancer

**DOI:** 10.3390/ijms22031223

**Published:** 2021-01-27

**Authors:** Jin Hur, Mithun Ghosh, Tae Heon Kim, Nahee Park, Kamal Pandey, Young Bin Cho, Sa Deok Hong, Nar Bahadur Katuwal, Minsil Kang, Hee Jung An, Yong Wha Moon

**Affiliations:** 1 Hematology and Oncology, Department of Internal Medicine, CHA Bundang Medical Center, CHA University, Seongnam 13496, Korea; hurjinz@naver.com (J.H.); mithunghoshmg@gmail.com (M.G.); Skgml0413@naver.com (N.P.); pkamal@chauniv.ac.kr (K.P.); mypeacemaker@hanmail.net (Y.B.C.); duggy126@gmail.com (S.D.H.); narbahadurkatwal@gmail.com (N.B.K.); rkdalstlf1097@gmail.com (M.K.); 2 Department of Biomedical Science, The Graduate School, CHA University, Seongnam 13496, Korea; 3 Department of Pathology, CHA Bundang Medical Center, CHA University, Seongnam 13496, Korea; Ice_t69@cha.ac.kr (T.H.K.); hjahn@cha.ac.kr (H.J.A.)

**Keywords:** chemotherapy-resistant ovarian cancer, belotecan, ataxia telangiectasia and Rad3-related inhibitor

## Abstract

Epithelial ovarian cancer remains the leading cause of mortality among all gynecologic malignancies owing to recurrence and ultimate development of chemotherapy resistance in the majority of patients. In the chemotherapy-resistant ovarian cancer preclinical model, we investigated whether AZD6738 (an ataxia telangiectasia and Rad3-related (ATR) inhibitor) could synergize with belotecan (a camptothecin analog and topoisomerase I inhibitor). In vitro, both chemotherapy-resistant and chemotherapy-sensitive ovarian cancer cell lines showed synergistic anti-proliferative activity with a combination treatment of belotecan and AZD6738. The combination also demonstrated synergistic tumor inhibition in mice with a chemotherapy-resistant cell line xenograft. Mechanistically, belotecan, a DNA-damaging agent, increased phospho-ATR (pATR) and phospho-Chk1 (pChk1) in consecutive order, indicating the activation of the DNA repair system. This consequently induced G2/M arrest in the cell cycle analysis. However, when AZD6738 was added to belotecan, pATR and pChk1 induced by belotecan alone were suppressed again. A cell cycle analysis in betotecan showed a sub-G1 increase as well as a G2/M decrease, representing the release of G2/M arrest and the induction of apoptosis. In ascites-derived primary cancer cells from both chemotherapy-sensitive and -resistant ovarian cancer patients, this combination was also synergistic, providing further support for our hypothesis. The combined administration of ATR inhibitor and belotecan proved to be synergistic in our preclinical model. This combination warrants further investigation in a clinical trial, with a particular aim of overcoming chemotherapy resistance in ovarian cancer.

## 1. Introduction

Epithelial ovarian cancer remains the leading cause of mortality among all gynecologic malignancies [1,2,3,4]. One of the main reasons why ovarian cancer has such a poor prognosis is attributed to delayed diagnosis because of the lack of symptoms until the late stage of the disease [4,5]. Although diagnosed at a late stage, ovarian cancer is usually surgically debulked, and the majority of patients respond to first-line platinum- and taxane-based chemotherapy [5]. However, the disease recurs after a disease-free period of several months to years in about 80% of patients [2,4]. In this recurrence setting, there is no gold-standard therapy as a second or further line of treatment. The most commonly used second-line treatments include retreatment with paclitaxel and/or carboplatin, pegylated liposomal doxorubicin, and topotecan [3,6,7]. However, chemotherapy resistance eventually develops in the majority of patients, which also leads to a poor prognosis.

Although systemic anticancer therapies, including poly (ADP-ribose) polymerase (PARP) inhibitors and bevacizumab, have recently been in progress in advanced ovarian cancer, their efficacies are very limited in the chemotherapy-resistant setting. In particular, PARP inhibitors such as olaparib [8], nirparib [9], and rucaparib [10] have provided tremendous survival benefits to platinum-responding patients, but PARP inhibitors do not work well in platinum-resistant patients compared to platinum-sensitive patients, even though the patients harbor the BReast CAncer gene (BRCA) mutation [11]. Although bevacizumab in combination with standard chemotherapy has improved progression-free survival by 3 months in patients with platinum-resistant ovarian cancer, overall survival was not prolonged [12]. Therefore, there is an urgent and medical unmet need for the development of a new therapeutic strategy in patients with chemotherapy-resistant ovarian cancer.

Topotecan, a topoisomerase I inhibitor and a water-soluble semisynthetic analog of the alkaloid camptothecin [4], has undergone extensive testing to define its role as a second-line agent for recurrent ovarian cancer, and has been approved by the US Food and Drug Administration (FDA) for that indication [13]. Belotecan (Camtobell; CKD602, 7-[2(*N*-isopropylamino) ethyl]-(20*S*)-camptothecin; Chong Kun Dang Pharmaceutical Corp., Seoul, Korea) is a new camptothecin analog in which a water-solubilizing group is introduced at position 7 of the B ring of camptothecin [14]. In preclinical studies, belotecan had a more potent topoisomerase I-inhibiting activity compared with topotecan [15]. Belotecan has demonstrated promising outcomes in recurrent ovarian cancer, achieving a 21–45% response rate when administered alone [15,16] and a 47–69% response rate when given in combination with platinum [17,18]. Thus, belotecan was approved for the treatment of recurrent ovarian cancer by the Korean FDA in 2003. Ataxia telangiectasia and Rad3-related protein (ATR) is one of the major DNA damage checkpoints, together with ataxia-telangiectasia-mutated protein (ATM) and DNA-dependent protein kinase (DNA-PK) [19]. ATM is primarily activated by DNA double-strand breaks (DSBs), whereas ATR responds to a much broader spectrum of DNA damage [19,20]. ATR inhibition prevents DNA damage checkpoint activation and disrupts DNA damage repair [21]. Therefore, when DNA-damaging stimulus affects DNA, simultaneous ATR inhibition leads to the accumulation of DNA single-strand breaks (SSBs) and DSBs, and, ultimately, the induction of tumor cell apoptosis. Taken together, these factors indicate that ATR inhibitors may sensitize tumor cells to DNA-damaging chemotherapies such as topoisomerase I inhibitors. Thus, we investigated the synergism of an AZD6738 in combination with belotecan in chemotherapy-resistant ovarian cancer.

## 2. Results

### 2.1. AZD6738 Synergizes with Belotecan for Cytotoxicity in Ovarian Cancer Cell Line

To investigate the potential synergistic anticancer activity of belotecan and AZD6738 in the ovarian cancer preclinical model, we first screened various ovarian cancer cell lines for the cytotoxicity of belotecan or AZD6738 alone. A list of various ovarian cancer cell lines is given in Supplementary Table S1 Among the chemotherapy-resistant cell lines such as SKpac-13 (paclitaxel-resistant) and A2780cis (cisplatin-resistant) that we screened, we selected SKpac-13 for further combination tests because SKpac-13 showed a higher IC_50_ (12 µM) for belotecan than A2780cis. Similarly, among the chemotherapy-sensitive cell lines—such as SNU-119, OVCAR-3, SKOV-3, and ID8—that we screened, we opted to use SNU-119 for further combination tests because SNU-119 showed the highest IC_50_ (140 nM) for belotecan among all chemotherapy-sensitive cell lines.

**Figure 1:**
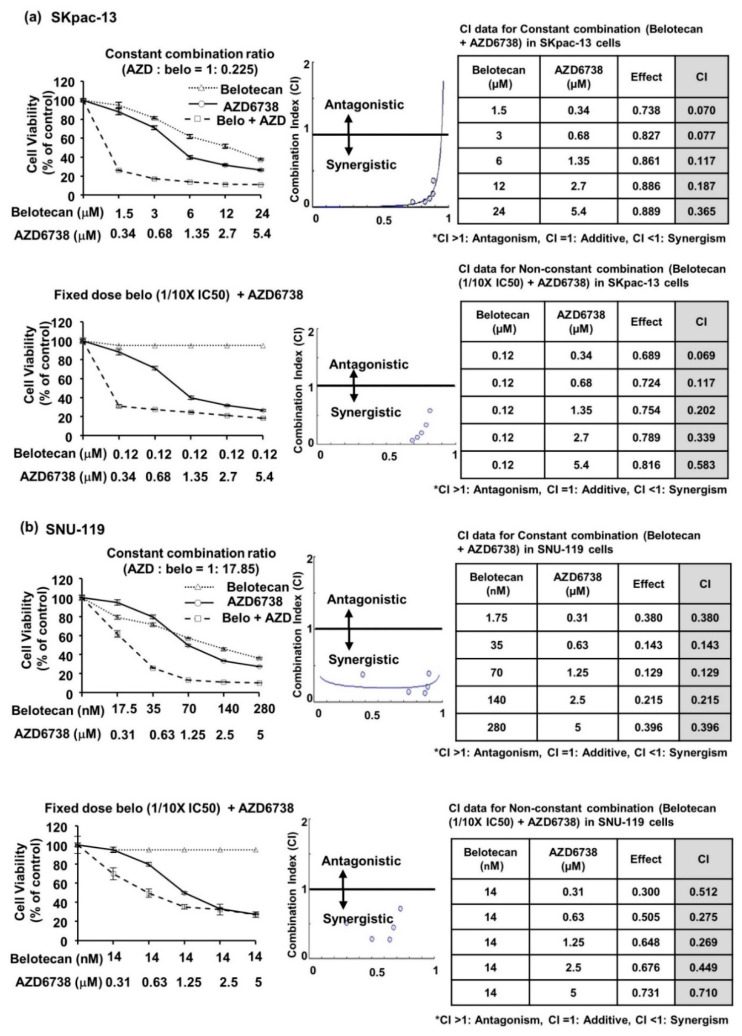
Thiazoyl blue tetrazolium bromide (MTT) assay demonstrated that AZD6738, an ataxia telangiectasia and Rad3-related protein (ATR) inhibitor, in combination with belotecan synergized in vitro epithelial ovarian cancer cell lines. (**a**,**b**) Combination of belotecan and AZD6738—in a constant combination ratio of both drugs or in a fixed dose of belotecan (1/10 IC_50_) and varying concentrations of AZD6738—demonstrated synergistic cytotoxicity in both SKpac-13 (**a**) and Seoul National University-119 (SNU-119) cells (**b**).

For the next step, we analyzed the combination cytotoxicity of belotecan and AZD6738 using a constant combination ratio of both drugs or a fixed dose of belotecan and various concentrations of AZD6738. In both paclitaxel-resistant (SKpac-13) and chemotherapy-sensitive (SNU-119) cells, a synergistic effect was observed over a wide range of combination concentrations (Figure 1a,b, Supplementary Figure S1b,c). In particular, a lower concentration of belotecan at 1/10 IC_50_ or 1/2 IC_50_ in combination with AZD6738 also showed synergism, suggesting the possibility of lowering the effective dosage of cytotoxic chemotherapy, belotecan, when combined with an ATR inhibitor.

**Figure 2:**
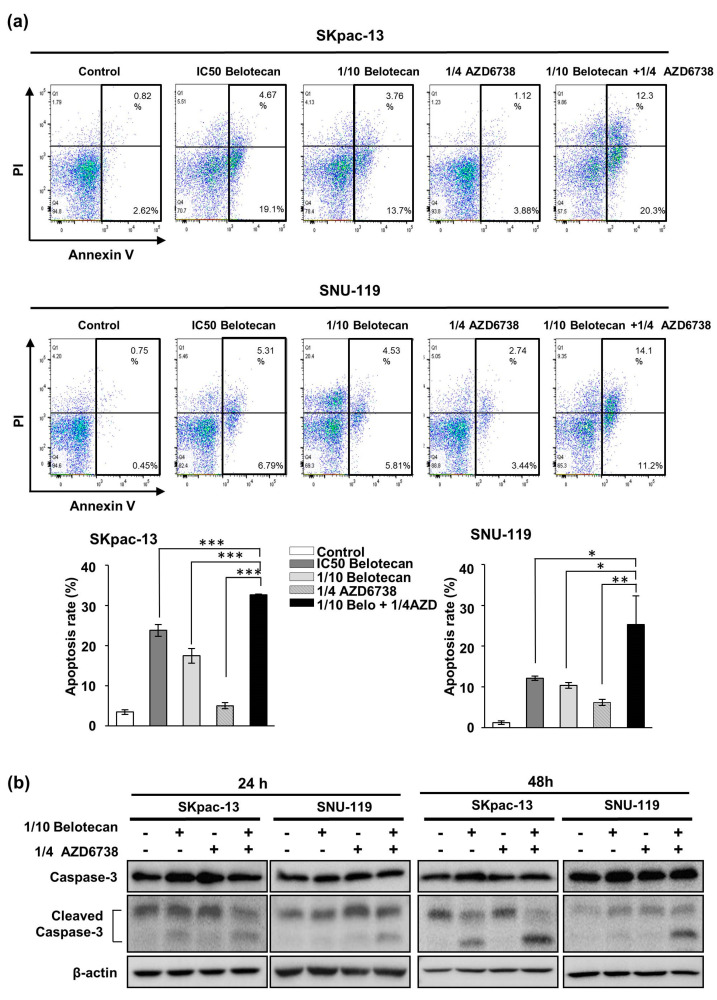
Synergistic effect of belotecan and AZD6738 on apoptosis. (**a**) Apoptosis assay demonstrated the synergistic effect of AZD6738 (1/4 IC_50_) combined with belotecan (1/10 IC_50_) in both SKpac-13 and SNU-119 cells. Data are presented as the mean ± standard deviation of triplicate experiments *p*-values were calculated by a student *t*-test, indicating * *p* < 0.05, ** *p* < 0.01, and *** *p* < 0.001. (**b**) Western blot showed that apoptosis marker, cleaved caspase-3, increased with the combination of both drugs, and beta actin was used as a loading control.

Furthermore, the apoptosis assay demonstrated that the combination of AZD6738 (1/4 IC_50_) and belotecan (1/10 IC_50_) synergistically increased apoptosis compared with a single treatment of each drug in SKpac-13 and SNU-119 cells, even though both drugs were combined in lower doses (1/4 IC_50_ AZD6738 plus 1/10 IC_50_ belotecan) compared with the effective dose (IC_50_ belotecan) as a single treatment (Figure 2a). In the Western blot assay, cleaved caspase-3 increased with the combination of both drugs, representing increased apoptosis (Figure 2b).

### 2.2. AZD6738 Induces DNA DSB by Suppressing DNA Damage Response Caused by Belotecan

To determine the mechanisms of synergism between belotecan and AZD6738 in chemotherapy-resistant and sensitive ovary cancer cells, we analyzed genes associated with DNA damage response using Western blot. Belotecan monotherapy, a DNA-damaging agent, induced phospho-ATR (pATR) and phospho-Chk1 (pChk1) in SKpac-13 and SNU-119 cells, indicating that the DNA damage repair system is activated by the belotecan-induced DNA damage of cancer cells [22]. Such a DNA repair mechanism might lessen the anticancer activity of belotecan when it is administered alone to patients.

**Figure 3:**
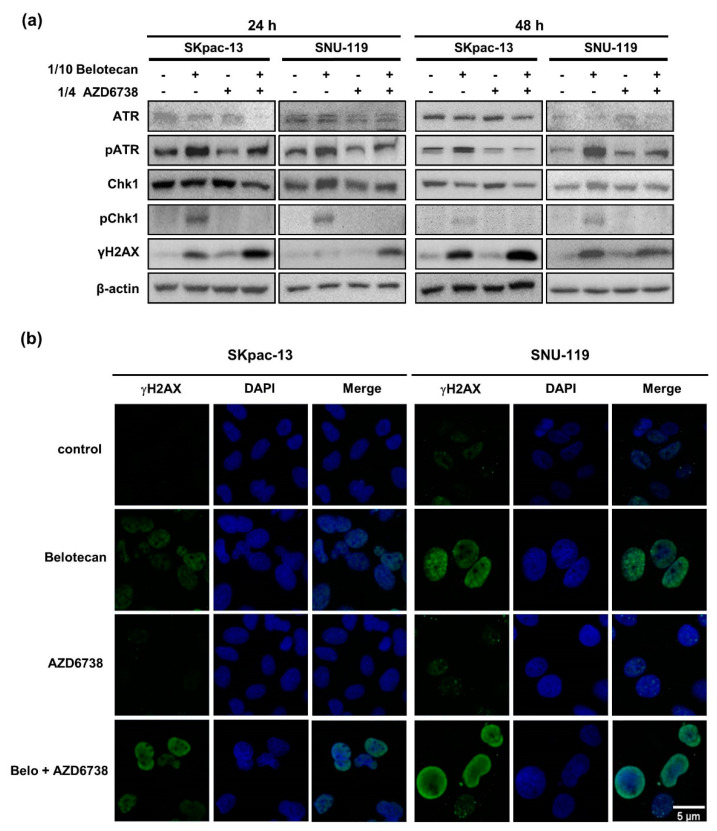
AZD6738 induces DNA double-strand break by suppressing the DNA damage response caused by belotecan. (**a**) Belotecan monotherapy-induced phospho-ATR (pATR) and phospho-Chk1 (pChk1) were suppressed again by the addition of AZD6738 to belotecan. The expression of the proteins was determined by a Western blot analysis and beta actin was used as a loading control. (**b**) Immunocytochemistry demonstrated that pan-nuclear γH2AX was significantly increased by the combination of AZD6738 and belotecan. The images were taken at 40× magnification and the scale bar was shown at 5 µm.

However, when AZD6738 was added to belotecan, pATR and pChk1 induced by belotecan alone were suppressed again. This indicates that the ATR inhibitor blocks the activation of the DNA damage repair system via the direct inhibition of ATR, one of the master regulators of DNA damage response. As a consequence, the DNA DSB marker, γ-H2AX, increased when AZD6738 was added to belotecan in SKpac-13 and SNU-119 cells (Figure 3a).

Immunocytochemistry staining demonstrated the formation of γ-H2AX foci in cells treated with belotecan alone, but not in AZD6738-treated cells. Furthermore, a pan-nuclear pattern of γ-H2AX—rather than γ-H2AX foci—was found in combination-treated cells, which was probably a result of the much greater DNA damage and DNA replication stress [23] induced by the combination treatment, considering that γ-H2AX foci, an indicator of double-strand DNA damage, can be progressive depending on the amount of DNA damage, ultimately reaching the widespread nuclear phosphorylation of histone H2AX, named the pan-nuclear pattern of γ-H2AX [23] (Figure 3b).

### 2.3. Mitotic Catastrophe Caused by AZD6738 in Combination with Belotecan May Account for Synergistic Mechanisms

**Figure 4:**
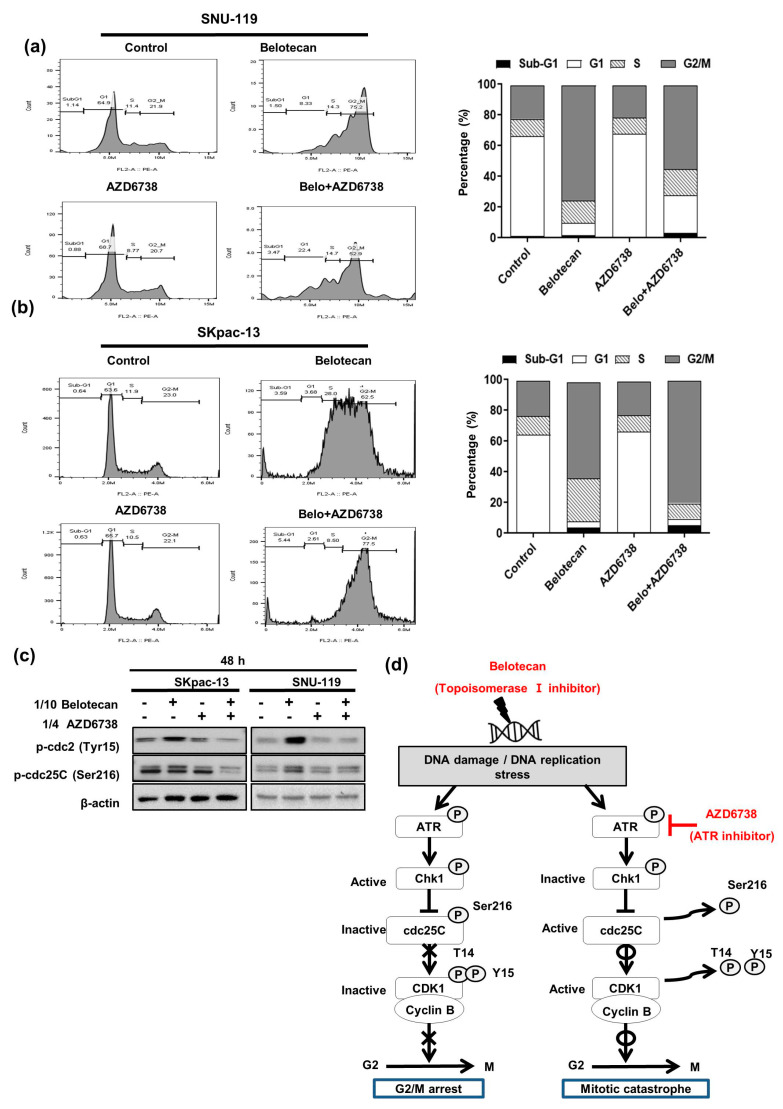
Mitotic catastrophe caused by AZD6738 in combination with belotecan may account for synergistic mechanisms. (**a**) Belotecan-induced G2/M arrest in the SNU-119 cells. (**b**) Belotecan-induced G2/M arrest in SKpac-13 cells. Addition of AZD6738 to belotecan led to more apoptosis, as indicated by the increased sub-G1 cell population compared to treatment with belotecan alone. (**c**) Downstream genes of Chk1, cdc25c, and phospho-CDK1 were increasingly phosphorylated with belotecan but were dephosphorylated again with the addition of AZD6738 to belotecan at 48 h. (**d**) Proposed synergistic mechanisms are depicted; belotecan-induced inhibitory phosphorylation of CDK1 causes mitotic exit, which is reversed by AZD6738, an ATR inhibitor, consequently allowing DNA-damaged cells to enter into mitosis, leading to mitotic catastrophe.

Next, in the cell cycle analysis we studied how cell cycle progression was affected by augmented DNA DSB induced by AZD6738 combined with belotecan. We observed more accumulated cells in the G2/M phase in the 48 h belotecan treatment (75.2% with a 1/10 IC_50_ concentration of belotecan) compared with the no-treatment control group (21.9%) using SNU-119 cells, suggesting increased G2/M arrest induced by belotecan. Here, the addition of AZD6738 to belotecan led to more apoptosis, as indicated by the simultaneous observation of increased sub-G1 cell population (3.47%) compared with the belotecan-only (1.50%) or AZD6738-only (0.88%) treatments (Figure 4a). Moreover, we also performed a cell cycle analysis in SKpac-13 cells and observed relevant results as compared to the SNU-119 cell line. Belotean (at 1/10 IC_50_ concentration) accumulated 62.5% of cells in the G2/M phase, whereas only 23% cells were accumulated in the control. Further, the combination treatment of AZD6738 and belotecan showed a higher number (77.5%) of cells at the G2/M phase as compared to the monotherapy. In addition, the cell number in the sub-G1 phase was increased (5.44%) after the combination treatment as compared to AZD6738 (0.63%) and belotecan (3.59%), which indicated that the combination treatment led to more apoptosis (Figure 4b).

To investigate how ATR inhibition regulates the cell cycle, as described above, we analyzed the downstream genes of Chk1, cdc25C, and cdc2 (also named as CDK1) using western blot, because phospho-Chk1 is known to suppress cdc25C by phosphorylation, and subsequently phospho-cdc25c is known to suppress the CDK1/cyclin B complex because of the inability of dephosphorylation to occur at Thr14 and Thy15 of CDK1 [24]. The expressions of phospho-cdc25C and phospho-CDK1 were increased with belotecan, but after some time they decreased again when AZD6738 was added to belotecan—that is, no change was observed at 24 h (Supplementary Figure S2a), but change was observed at 48 h (Figure 4c)—indicating that alteration in downstream genes may take time.

To sum up what we have observed about overall synergistic mechanisms so far, belotecan induces DNA damage, phosphorylating ATR, and subsequently phosphorylating cdc25C, turning off phosphatase activity of cdc25c. Finally, inactive cdc25C leads to inhibitory phosphorylation at the Thr14 and Thy15 sites of CDK1 as a form of the CDK1/cyclin B complex, resulting in mitotic exit—that is, G2/M arrest. On the contrary, ATR inhibition by the addition of AZD6738 to belotecan causes the reverse process. Ultimately, dephosphorylation at Thr14 and Thy15 of CDK1 allows DNA-damaged cells to enter into mitosis, leading to mitotic catastrophe (Figure 4d).

### 2.4. AZD6738 and Belotecan Combination Effectively Suppresses Tumor Growth in a Xenograft Model

To test the in vivo synergistic effect of combined AZD6738 and belotecan treatment, we used a nude mice model xenografted with SKpac-13 cells. Initially, we performed a preliminary in vivo study to test the toxicity of the combined AZD6738 and belotecan treatment. Treatments of AZD6738 at 30 or 40 mg/kg (daily, oral gavage) in combination with belotecan 20 mg/kg (every 4 days, intraperitoneally) were administered. A weight loss of about 2.5 g per mouse was observed in both groups. Moreover, two of six mice treated with AZD6738 30 mg/kg and three of five mice treated with AZD6738 40 mg/kg died 35 days after the drug administration.

**Figure 5:**
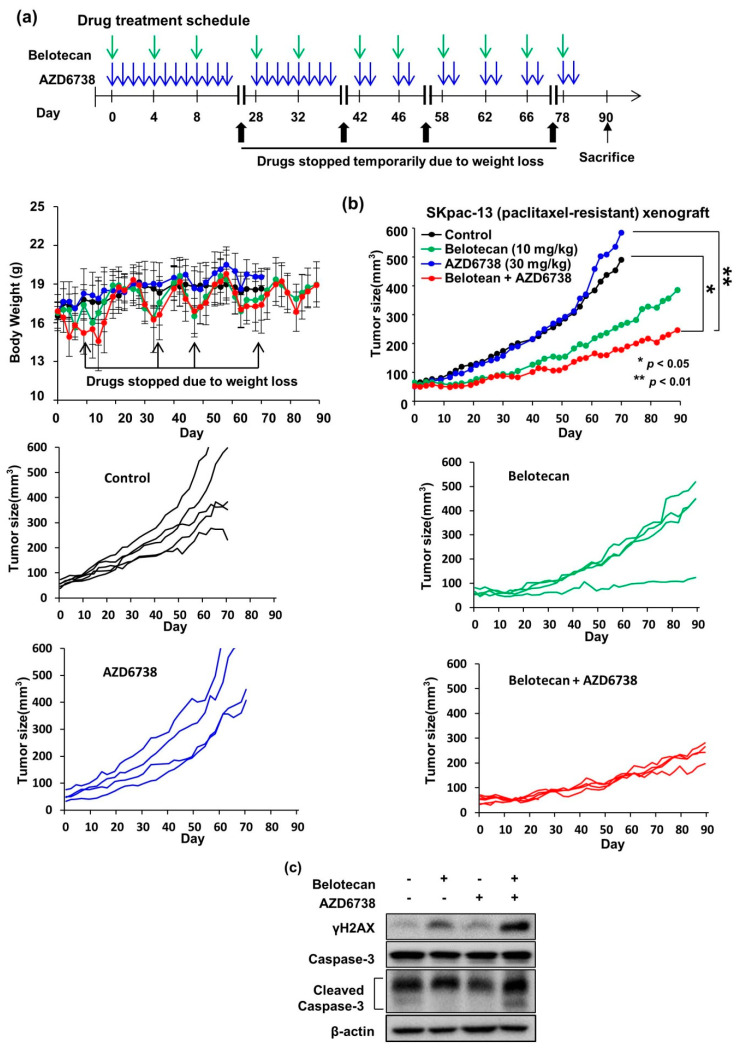
AZD6738 an ATR inhibitor in combination with belotecan demonstrate synergism in vivo using epithelial ovarian cancer cell line xenograft. (**a**) Drug treatment schedule and body weight change. AZD6738 30mg/kg was administered via oral gavage daily and belotecan 10 mg/kg was injected intraperitoneally every 4 days for single or combination dosages. In this combination dosage, mice lost weight by an average of 1.7 g on day 10 and appeared very sick. Then, both drugs were temporarily stopped until body weight was recovered. On day 28, both drugs were started again with the same schedule and dosages, but were stopped again with the re-occurrence of similar toxic effects on day 35. Starting on day 42, we started dosing again with a different dosing schedule of AZD6738 from daily dosing to 2 days on/2 days off. After intermittent dosing, the weight loss was reduced to an average of 1.1 g per mouse. (**b**) Combined AZD6738 30 mg/kg (oral gavage, daily but changed to 2 days on and 2 days off from day 42) and belotecan suppressed tumor growth more effectively than the no-treatment control or each single treatment. In the right panel, the average tumor sizes of all mice in each group are depicted. In the right and left panel, the tumor sizes of individual mice in each group are depicted. The tumor size of individual mice in a graph (combination group) depicted for 6 mice. At the beginning, 6 curves can be seen, but only four curves are visible at the end as two mice died in the early stage. *p* values were calculated using student t-test, where * *p* < 0.05, ** *p* < 0.01. Data are presented as mean ± standard deviation. (**c**) Western blot using SKpac-13xenograft after 90 days of treatment. Beta actin was used as a loading control.

Then, we selected AZD6738 at 30 mg/kg (daily, oral gavage) and belotecan at 10 mg/kg (every 4 days, intraperitoneally) for single or combination dosages. However, two mice died in the combination group between 10 and 23 days and the remaining lost weight at an average of 1.7 g. Therefore, starting on day 42 we changed the dosing schedule of AZD6738 from daily dosing to 2 days on and 2 days off by referencing the human phase I trial of combined AZD6738 (7 days on/21 days off) and olaparib [25]. After intermittent dosing, the weight loss was reduced to an average of 1.1 g per mouse (Figure 5a).

Regarding efficacy, on day 70 after drug administration the tumor size reached 500–600 mm^3^ in the no-treatment control and AZD6738 groups, which was significantly larger than that in the belotecan group (*p* = 0.022) or the combination group (*p* = 0.007). Therefore, mice in the no-treatment control and AZD6738 groups were sacrificed at this time point. The combined treatment group showed a trend toward greater tumor growth inhibition compared with belotecan or AZD6738 alone (*p* > 0.05) (Figure 5b). Western blot using SKpac-13 xenograft after 90 days of treatment demonstrated that pATR and cleaved caspase-3 were increased at a higher rate in the combination group compared with the single treatment groups, supporting the in vitro data (Figure 5c).

### 2.5. Analysis of Patient Fluid Samples Supports Synergism of AZD6738, ATR Inhibitor, and Belotecan in Ovarian Cancer

**Figure 6:**
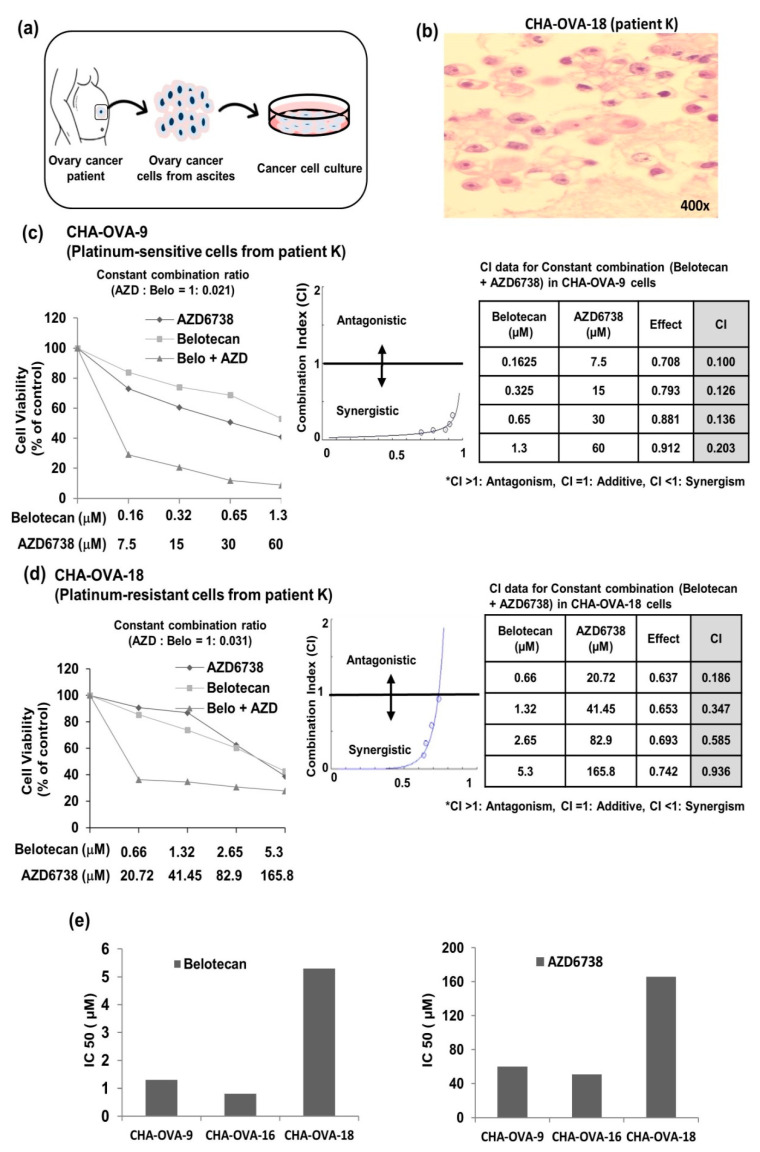
AZD6738, an ATR inhibitor, in combination with belotecan demonstrates synergism in vitro primary ovarian cancer cells: (**a**) schematic diagram for the collection of ascitic fluid from ovarian cancer patients and the culture of primary cancer cells. (**b**) Representative image of H&E staining, which confirmed that the CHA-OVA-18 cells were ovarian cancer cells. (**c**,**d**) Combination of belotecan and AZD6738—in a constant combination ratio of both drugs—demonstrated synergistic cytotoxicity in both CHA-OVA-9 (platinum-sensitive) (**c**) and CHA-OVA-18 (platinum-resistant) primary ovarian cancer cells (**d**). (**e**) Bar graph representing the comparison of IC_50_ among the three primary ovarian cancer cells—namely CHA-OVA-9, CHA-OVA-16, and CHA-OVA-18.

Primary ovarian cancer cells derived from the ascitic fluid sample of ovarian cancer patients (Figure 6a) were used for an ex vivo assay (Figure 6c,d and Supplementary Figure S2b). CHA-OVA-9 and CHA-OVA-16 cells were collected from chemotherapy-naïve patients, whereas CHA-OVA-18 cells were collected from a chemotherapy-treated patient. Results of the MTT assay showed IC_50_ values of 1.3 µM with belotecan alone and 60 µM with AZD6738 alone for CHA-OVA-9 cells (Figure 6c) and 5.3 µM belotecan and 165.8 µM AZD6738 for CHA-OVA-18 cells (Figure 6d). Moreover, the IC_50_ in CHA-OVA-16 cells was 0.81 µM and 50.73 µM for belotecan and AZD6738, respectively (Figure 2b). Meanwhile, the combination treatment markedly decreased the IC_50_ values to a large extent in all the three primary ovarian cancer cells, further supporting the synergistic anti-proliferative effect of AZD6738 and belotecan. More interestingly, the CHA-OVA-9 (platinum-sensitive) and CHA-OVA-18 (platinum-resistant) cells are from the same patient and CHA-OVA-18 was considered platinum-resistant because it was collected after recurrence with only 5 months of platinum-free interval, following the paclitaxel/carboplatin/bevacizumab treatment. The MTT data also supported our consideration that CHA-OVA-18 cells have platinum resistance, because the IC_50_ values were higher in the CHA-OVA-18 cells than in the CHA-OVA-9 cells (Figure 6e). In addition, it was demonstrated that the combination treatment of belotecan and AZD6738 showed a synergistic effect even in platinum-resistant primary ovarian cancer cell at a very low concentration. The CHA-OVA-18 cells were ovarian cancer cells, as confirmed by H & E stain (Figure 6b).

## 3. Discussion

Considering that recurrent ovarian cancer is an incurable disease, the enhancement of chemotherapeutic activity is regarded as an urgent and unmet medical need in the recurrent setting as well as in the adjuvant setting. We have demonstrated that ATR inhibitor potentiated the activity of belotecan, which is one of the mainstay chemotherapies in recurrent ovarian cancer. Furthermore, the combination treatment presented in this study also showed synergism even in the chemotherapy-resistant ovarian cancer preclinical model, suggesting the possibility of overcoming resistance.

Previously, other researchers have reported that ATR inhibitors increased the anticancer activity of various DNA-damaging or DNA synthesis-inhibiting therapies, such as topotecan [26], cisplatin [26,27], gemcitabine [26,27], and PARP inhibitor [26,28]. Our results are in parallel with the findings of these reports. Specifically, in this study we focused on the ovarian cancer model because ovarian cancer is a neoplasm strongly linked to defects in DNA repair mechanisms, by which DNA-damaging anticancer therapies such as platinum, topoisomerase inhibitors, anthracyclines, or PARP inhibitors [29] are clinically more effective and widely used. In addition, uniquely in this study belotecan, a novel topoisomerase I inhibitor, which is a Korean FDA-approved anticancer drug in ovarian or small cell lung cancer, was tested in combination with ATR inhibitor.

Mechanistically, the inhibition of ATR by AZD6738 led to mitotic catastrophe via driving DNA-damaged cells by belotecan into the mitotic phase without repairing damaged DNA. This synergistic mechanism of AZD6738 combined with belotecan is quite similar to that mentioned in a previous study of ATR inhibitor in combination with PARP inhibitor [28]. We also demonstrated that ATR inhibition regulated the cell cycle via downstream genes, such as Chk1, cdc25C, and cdc2 (also known as CDK1), on the point of inducing mitotic catastrophe in DNA-damaged cells. Although DNA checkpoints other than ATR (i.e., ATM or DNA-PK) can be activated by DNA-damaging stimuli such as belotecan, we consider that adding ATR inhibitor to belotecan might be mechanistically more beneficial based on the ATR responsiveness to a much broader spectrum of DNA damage [19,20], as mentioned earlier.

In terms of the toxicity of this combination, the continuous dosing of AZD6738 and every 4-day dosing of belotecan caused severe toxicity in the mouse model. This might be attributable to the persistence of DNA damage in normal cells, which results from the continuous inhibition of ATR. When we changed the dosing schedule of AZD6738 to an intermittent mode of 2 days on/2 days off, this combination was tolerated by the mice. If a clinical trial is designed with this combination, we recommend that AZD6738 be administered intermittently with belotecan. Furthermore, in the previous phase I trial of combined AZD6738 and olaparib, AZD6738 was safely given in an intermittent mode of 7 days on/21 days off [25]. There have been one published and three ongoing phase I trials with another ATR inhibitor, VX-970 (M6620; Vertex Pharmaceuticals, Boston, MA, USA), in combination with a topoisomerase inhibitor, topotecan or irinotecan, as listed in Supplementary Table S2. The combination of AZD6738 and belotecan has never been tested in a clinical trial. Further, our combination may benefit platinum-resistant ovarian cancer patients based on the preclinical synergism shown in this study. In a future clinical trial design, in chemotherapy-resistant ovarian cancer patients, belotecan dosage might be reduced with an expectation of similar or higher anticancer activity in combination with AZD6738 based on the stronger anticancer effect by combining even lower dosages of both drugs than the IC_50_ dosage of belotecan alone in our preclinical model.

In conclusion, the combined administration of ATR inhibitor and belotecan was shown to be synergistic in both the chemotherapy-resistant ovarian cancer preclinical model and the chemotherapy-sensitive model. This combination warrants further investigation in a clinical trial with the particular aim of overcoming chemotherapy resistance in ovarian cancer.

## 4. Materials and Methods

### 4.1. Reagents

Belotecan was provided by Chong Kun Dang Pharmaceutical Corp (Seoul, Korea), and dissolved in dimethyl sulfoxide (DMSO) (Biosesang, Gyeonggi-do, Korea) at 10 mg/mL, which is the highest concentration used in vitro, then stored as the stock solution in aliquots at −20 °C until use. AZD6738 was provided by AstraZeneca (Macclesfield, Cheshire, UK) and initially dissolved in DMSO. Meanwhile, paclitaxel was purchased from Selleck Chemicals (Houston, TX, USA).

### 4.2. Cell Line and Cell Culture

The ovarian cancer cell lines OVCAR-3 and SKOV3 were purchased from the American Type Culture Collection (Manassas, VA, USA), whereas SNU-119 was purchased from Korean Cell Line Bank (Seoul, Korea). A2780cis was obtained from the European Collection of Animal Cell Cultures (Porton Down, Salisbury, UK). The ID8 murine ovarian epithelial cancer cell line was provided by the University of Kansas Medical Center (Kansas City, KS, USA). SKpac-13 was donated by Hee Jung An laboratory at CHA Bundang Medical Center (Seongnam, Korea) [30]. Each cell line was maintained in RPMI (Roswell Park Memorial Institute) 1640 medium (Welgene, Daegue, Korea), supplemented with 10% heat-inactivated fetal bovine serum (FBS; Welgene, Daegue, Korea), 100 µg/mL streptomycin (Welgene, Daegue, Korea), and 100 IU/mL penicillin (Welgene, Daegue, Korea) in a humidified incubator at 37 °C with a 5% CO_2_ atmosphere. To transfer or passage the cell lines, each confluent monolayer was washed with phosphate-buffered saline (PBS; Welgene, Daegue, Korea) and detached with a 0.05% trypsin/0.02% ethylenediaminetetraacetic acid (EDTA) solution (Welgene, Daegue, Korea).

### 4.3. Cell Viability Assay

Cell proliferation was measured using the thiazoyl blue tetrazolium bromide (MTT; Sigma, St. Louis, MO, USA) colorimetric assay. Cells at a density of 2.5–3 × 10^3^ cells/well in 100 µL RPMI with 10% FBS were added to the wells of a 96-well plate and incubated overnight. The cells were treated with different concentrations of belotecan alone, AZD6738 alone, and their combination for 48 h. After drug treatment, the MTT solution was added to each well, and the plates were incubated for 4 h at 37 °C; then, the medium was removed. After dissolving the formazan crystals that were formed in 100 µL of DMSO, the absorbance of each plate was measured at 570 nm using a Multiskan GO Microplate Spectrophotometer (Thermo Fisher Scientific, Vantaa, Finland). The absorbance and half-maximal inhibitory concentration (IC_50_) and combination index values of belotecan or AZD6738 were analyzed using the CompuSyn software (ComboSyn, Inc., Paramus, NJ, USA).

### 4.4. Apoptosis Assay

For the apoptosis assay, SKpac-13 and SNU-119 cells were harvested 48 h after drug treatment. In brief, cells were washed in PBS and resuspended with 500 µL of Annexin V 1× binding buffer. Cells were stained with Annexin V-APC (BioLegend, San Diego, CA, USA) and propidium iodide (PI) (Invitrogen, Carlsbad, CA, USA) and incubated for 20 min at room temperature in the dark. Stained cells were washed with cold PBS two times and analyzed for apoptosis with flow cytometry (Beckman Coulter Cytoflex, Indianapolis, IN, USA).

### 4.5. Western Blot Analysis

Protein samples were prepared by lysing cells in radioimmunoprecipitation assay (RIPA) buffer (Thermo Scientific, Rockford, IL 61101, USA) containing protease inhibitor (Roche, Basel, Switzerland) and phosphatase inhibitor cocktail (Thermo Scientific, Rockford, IL 61101, USA) and were stored at 4 °C for 1 h. After centrifugation (14,000 rpm) at 4 °C for 15 min, the supernatant was collected. To extract protein from tumor tissue, tumors were dissected and wash briefly wash with 1X PBS. Tumor tissues were cut into smaller pieces and transferred to a homogenizer and added RIPA buffer containing protease inhibitor and phosphatase inhibitor cocktail. They were homogenized thoroughly and the sample was kept in ice for 1 h and vortexed occasionally. The samples were then centrifuged (same condition as mentioned above) and supernatants were collected. Proteins in whole-cell lysate and tumor tissue lysate (30 µg) were separated using sodium dodecyl sulfate-polyacrylamide gel electrophoresis (SDS-PAGE), and electrotransferred onto polyvinylidenedifluoride (PVDF) (Merck KGaA, Darmstadt, Germany) membranes. Membranes were blocked for 1 h in Tris-buffered saline containing 5% milk and 0.1% Tween 20 at room temperature and probed with the following primary antibodies: phospho-ATR, ATR (Thr1989), phospho-Chk1 (Ser345), Chk1, caspase-3/cleaved caspase-3, phospho-cdc25C (Ser216), phospho-cdc2 (Tyr15), phospho-histone H2AX (Ser139/Tyr142), and β-actin. A list of antibodies is given in Supplementary Table S3.

### 4.6. Immunocytochemistry

SKpac-13 and SNU-119 cells were fixed with 4% paraformaldehyde for 30 min at room temperature. Before primary antibody staining, the cells were incubated for 1 h in a blocking solution consisting of 5% donkey serum with 10% Triton X-100 in PBS. After that, the cells were incubated with phospho-histone H2AX (Ser139/Tyr142) polyclonal antibody (Cell Signaling Technology, Danvers, MA, USA) diluted in 1:100 in a blocking solution for 1 h at room temperature. Next, the cells were washed in PBS, and then incubated with secondary antibodies (Alexa Fluor 488 conjugated) diluted in 1:250 in a blocking solution for 1 h in the dark at room temperature. Finally, the cells were washed with PBS and coverslipped with the Flouoro-shield mounting medium containing 4′,6-diamidino-2-phenylindole (DAPI; abcam, Cambridge, United Kingdom), and then examined using a fluorescence microscope LSM 880 (Zeiss, Oberkochen, Germany).

### 4.7. Cell Cycle Analysis

The cells were plated at a density of 3 × 10^5^ cells/well in 60 mm culture dishes. The cells were treated with drugs for 24 h. Then cells were harvested, washed twice in ice-cold PBS, and then fixed in 70% ethanol at −20 °C for a minimum of 1 h or overnight. Next, they were washed with PBS, and incubated with 100 µg/mL RNase A (Sigma-Aldrich, St. Louis, MO, USA) and 50 µg/mL PI (Invitrogen, Carlsbad, California, USA) at 37 °C for 15 min. Finally, the cells were washed with PBS and analyzed using flow cytometry (Beckman Coulter Cytoflex, Indianapolis, IN, USA).

### 4.8. In Vivo Study

For the tumor xenograft experiments, 5-week-old female BALB/c-nude mice (18–20 g) were purchased from Orient Bio Inc. (Seongnam, Korea). All the mice were housed in a specific-pathogen-free animal facility under controlled temperature (24 ± 3 °C) and 12 h light/dark cycle with free access to food and water at CHA University (Seongnam, Korea). Animals were acclimatized to the environment for at least 1 week prior to their use in the experiment. All animal procedures were followed according to the approved protocol by the Institutional Animal Care and Use Committee (IACUC) of CHA University (IACUC190036). SKpac-13 (5 × 10^6^) cells were inoculated subcutaneously in the right flank of the mouse. When the tumor volume reached about 50–70 mm^3^, the mice were randomized into four groups. The experiment was started with the following number if mice in each group (control = 5, Belotecan = 4, AZD6738 = 4 and combination = 6). Initially we performed a preliminary experiment to determine the drug toxicity and selected AZD6738 30 mg/kg and belotecan 10 mg/kg for further experiment [31,32,33,34,35].Mice were injected with PBS (group 1), belotecan (10 mg/kg in captisol (CyDex Pharmaceuticals, Inc. Kansas, USA); every 4 days, intraperitoneally; group 2), AZD6738 (30 mg/kg in captisol; daily, oral gavage; group 3), and combined belotecan and AZD6738 (group 4). Starting on day 42, the dosing schedule of AZD6738 was changed to an intermittent mode of 2 days on/2 days off because of toxicity concerns. Tumor sizes were measured using a vernier caliper three times a week and calculated (tumor length^2^ × tumor width^2^ × 0.5). In addition, body weight was assessed three times a week. After 70 days, mice in the control group and the AZD6738 group were sacrificed because the predetermined tumor size had been reached, whereas mice in the belotecan group and the combination group were sacrificed after 90 days according to the animal experimental guidelines. The xenografted tumors were then excised and preserved for further analysis.

### 4.9. Clinical Sample

Ascitic fluid sample was collected from the patients with epithelial ovarian cancer whose histology was high-grade serous carcinoma. Approximately 200 mL of fluid was first filtered through a 100 µM cell strainer and centrifuged at 400 *g* at 40 °C for 10 min, then the precipitated cells were washed with PBS. Mononuclear cells were isolated after Ficoll-Paque Plus (GE Healthcare, Sigma-Aldrich, Inc.) density gradient centrifugation at 400 *g* for 30 min. Cells were washed twice with PBS at 100 g for 10 min and mixed 1:2 with RPMI-1640 resuspended with 15% FBS and 1% penicillin/streptomycin and transferred to multiple T-75 culture flasks. Cells were grown until 70–80% confluency, after which they were subcultured. Passage two or three was used for the cell viability assay. The collection of patient samples was approved by the Institutional Review Board (IRB) of the CHA Bundang Medical Center (IRB number: 2016-03-037-012).

### 4.10. H&E Staining of Cell Block

CHA-OVA cells were cultured with RPMI-1640. When the plates were fully confluent, cells were collected by trypsinization. Next, the cells were centrifuged and the cell pellets were placed at the center of the plate and surrounded by 1.5% agarose gel. When the gel became solid, the area of the gel containing the cell pellets were cut and incubated in 4% paraformaldehyde for fixation. Lastly, the cell blocks were sent to the histology lab for preparation of paraffin block. To perform hematoxylin and eosin staining, 5 µM sections were prepared from the cell block by sectioning with a microtome and then placed on slides. Next, the slides were deparaffinized and washed the xylene by 100% alcohol. Next, the slides were stained with hematoxylin (DAKO, Carpinteria, CA, USA) for 7 min, and after neutralization eosin (Sigma-Aldrich, St. Louis, MO, USA) was added and they were stained for 5 min. Finally, after the dehydration process the coverslips were mounted and examined under a light microscope.

### 4.11. Statistical Analysis

Statistical analysis was performed using SPSS version 19.0 (IBM SPSS Statistics 19.0; IBM, Armonk, NY, USA). Student’s T test was used to determine the significance of the differences between each group. All the *p* values were two-sided, and *p* values of less than 0.05 were considered statistically significant.

## Supplementary Materials

Supplementary Materials can be found at https://www.mdpi.com/1422-0067/22/3/1223/s1. Figure S1. (a) Chemical structure of belotecan and AZD6738. (b,c) MTT assay indicated that various concentration of AZD6738, in combination with IC50 and half IC50 of belotecan synergize in Skpac-13 (b) and SNU-119 (c) epithelial ovarian cancer cell lines. Figure S2. (a) Downstream genes of Chk1, cdc25c, and phospho-CDK1 were increasingly phosphorylated with belotecan at 24 h. The expression of the proteins was determined by the western blot analysis and beta actin was used as loading control. (b) MTT assay demonstrated that Combination of belotecan and AZD6738—in a constant combination ratio of both drugs—demonstrated synergistic cytotoxicity in both CHA-OVA-16 primary ovarian cancer cells. Table S1. Various ovary cancer cell lines were screened for cytotoxicity of belotecan or AZD6738 alone. Table S2. Published and ongoing clinical trials of combined ATR inhibitor and topoisomerase inhibitor. Table S3. Primary antibodies used for western blot or Immunocytochemistry.
