# Supplementary material for: Synergism of AZD6738, an ATR Inhibitor, in Combination with Belotecan, a Camptothecin Analogue, in Chemotherapy-Resistant Ovarian Cancer
Source: Int J Mol Sci. 2021 Jan 27;22(3):1223. doi: 10.3390/ijms22031223 (PMC7865398; doi:10.3390/ijms22031223)
Supplement: Supplementary file 1 [file ijms-22-01223-s001.zip › ijms-1049612-supp-final-layout/Additional information_Uncropped western images.pdf]

International Journal of *Molecular Sciences*

Article

Synergism of AZD6738, ATR inhibitor in combination with belotecan, Camptothecin analogue, in chemotherapy-resistant ovarian cancer

Jin Hur[1, 2], Mithun Ghosh[1,2], Tae Heon Kim[3], Nahee Park[1], Kamal Pandey[1], Young Bin Cho[1], Sa Deok Hong[1,2], Nar Bahadur Katuwal[1,2], Minsil Kang[1], Hee Jung An[3], Yong Wha Moon[1*]

[1]Hematology and Oncology, Department of Internal Medicine, CHA Bundang Medical Center, CHA University, Seongnam, Korea;
hurjinz@naver.com (J.H.); mithunghoshmg@gmail.com (M.G.); Skgml0413@naver.com (N.P.); pkamal@chauniv.ac.kr (K.P.); mypeacemaker@hanmail.net (Y.B.C.); duggy126@gmail.com (S.D.H.); narbahadurkatwal@gmail.com (N.B.K.); rkdalstlf1097@gmail.com (M.K.)
[2]Department of Biomedical Science, The Graduate School, CHA University, Seongnam, Korea;
Ice_t69@cha.ac.kr (T.H.K.); hjahn@cha.ac.kr (H.J.A.)
[3]Department of Pathology, CHA Bundang Medical Center, CHA University, Seongnam, Korea
*Correspondence: ymoon@cha.ac.kr (Y.W.M.); Tel: +82-31-780-3436; Fax: +82-31-780-3929

Corresponding Author:
Yong Wha Moon, MD, PhD
Hematology and Oncology, Department of Internal Medicine, CHA Bundang Medical Center, CHA University, Seongnam-si, Gyeonggi-do, 463-712, Korea, Tel: +82-31-780-3436, Fax: +82-31-780-3929, E-mail: ymoon@cha.ac.kr

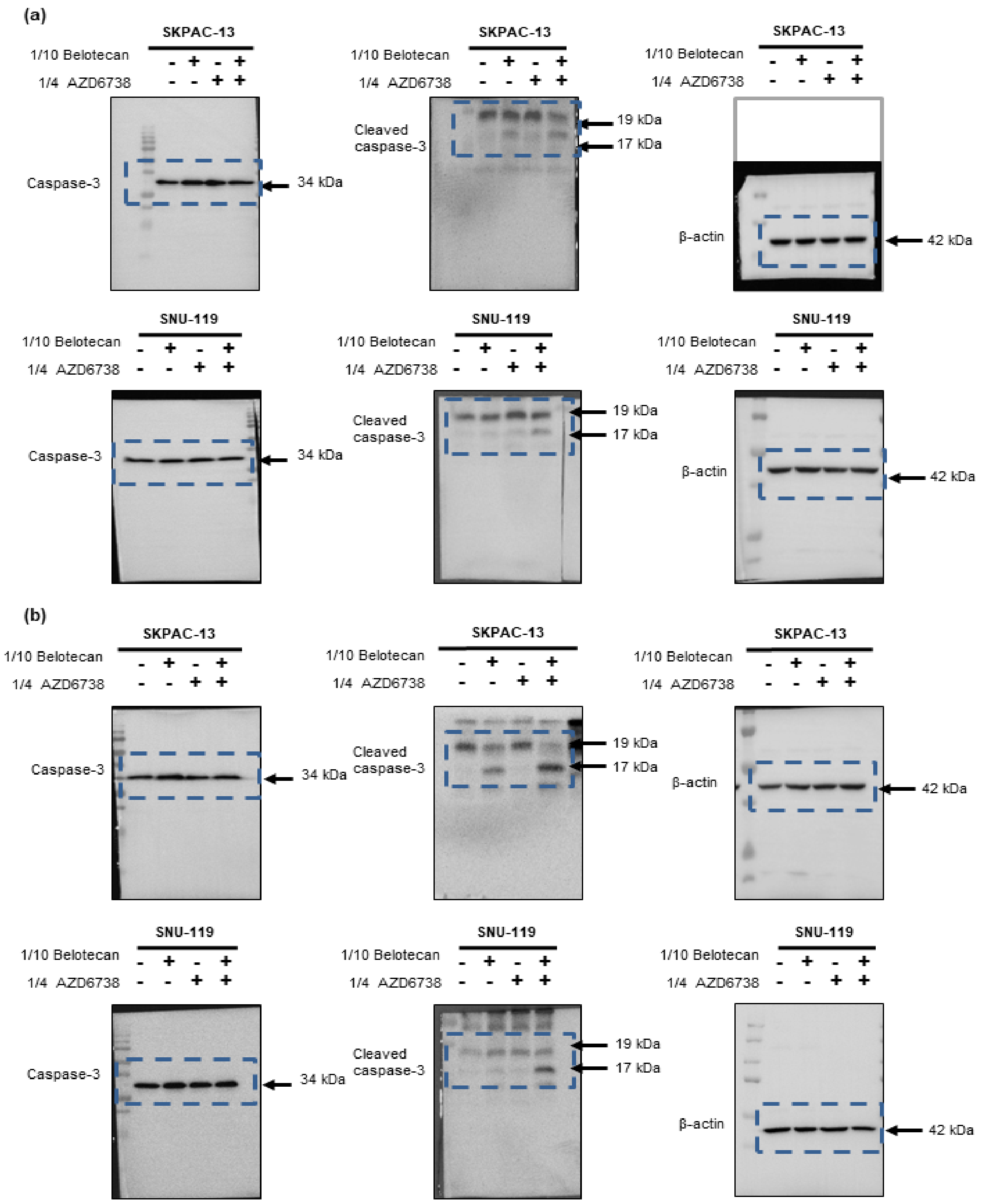

Raw western blot data of the cropped images shown in figure 2b. Western blotting of Caspase-3, Cleaved Caspase-3 from Skpac-13 cells and SNU-119 cells. Each cells were treated with belotecan, AZD6738 and combination of both for (a) 24h and (b) 48h. β-actin was used as loading control.

(a)

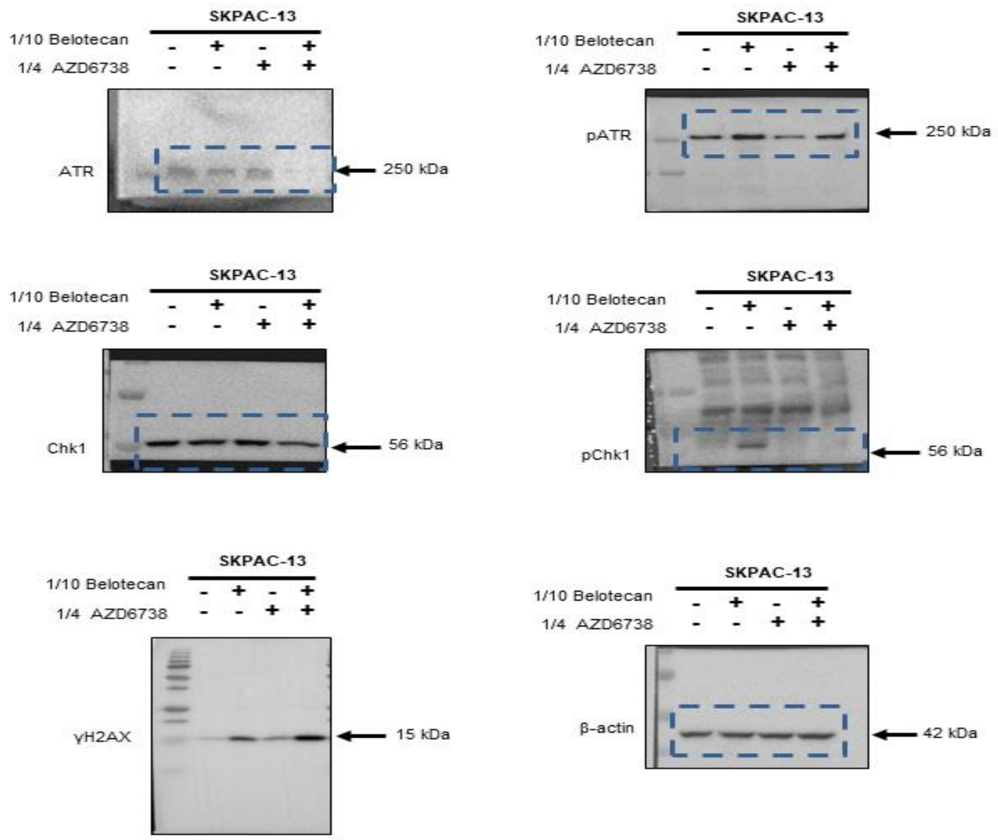
SKPAC-13
1/10 Belotecan
1/4 AZD6738
ATR
250 kDa
pATR
250 kDa
Chk1
56 kDa
pChk1
56 kDa
γH2AX
15 kDa
β-actin
42 kDa

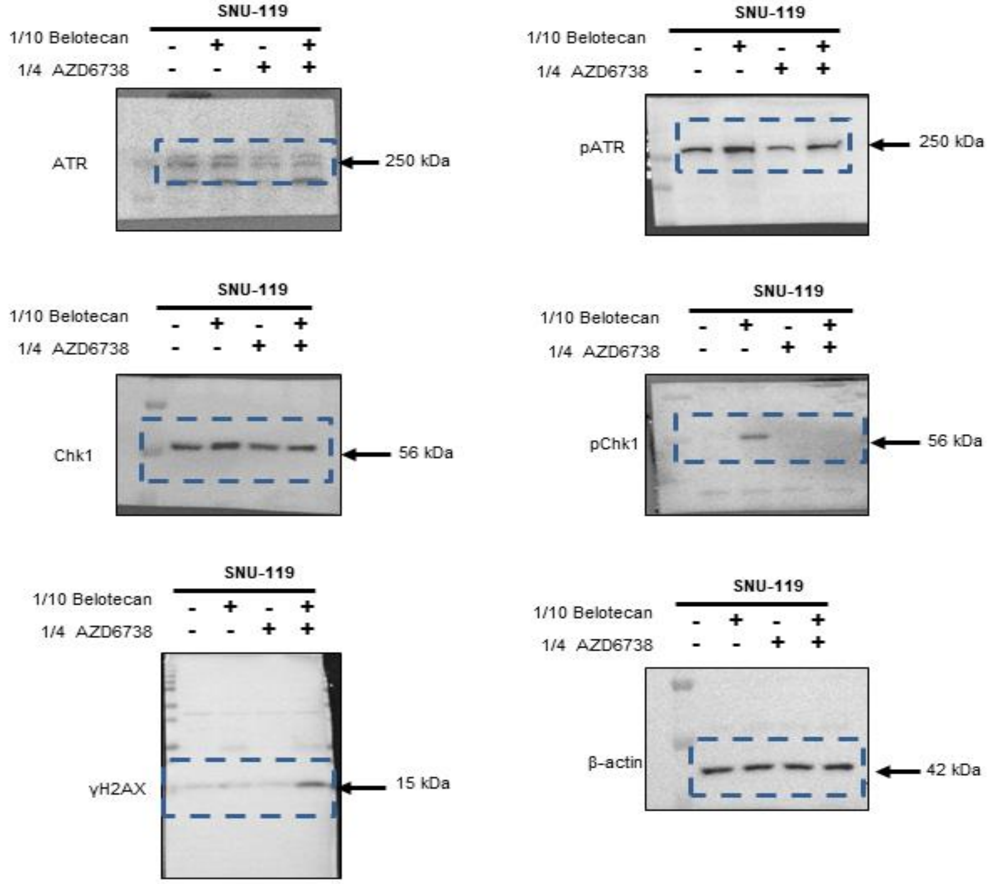
SNU-119
1/10 Belotecan
1/4 AZD6738
ATR
250 kDa
pATR
250 kDa
Chk1
56 kDa
pChk1
56 kDa
γH2AX
15 kDa
β-actin
42 kDa

(b)

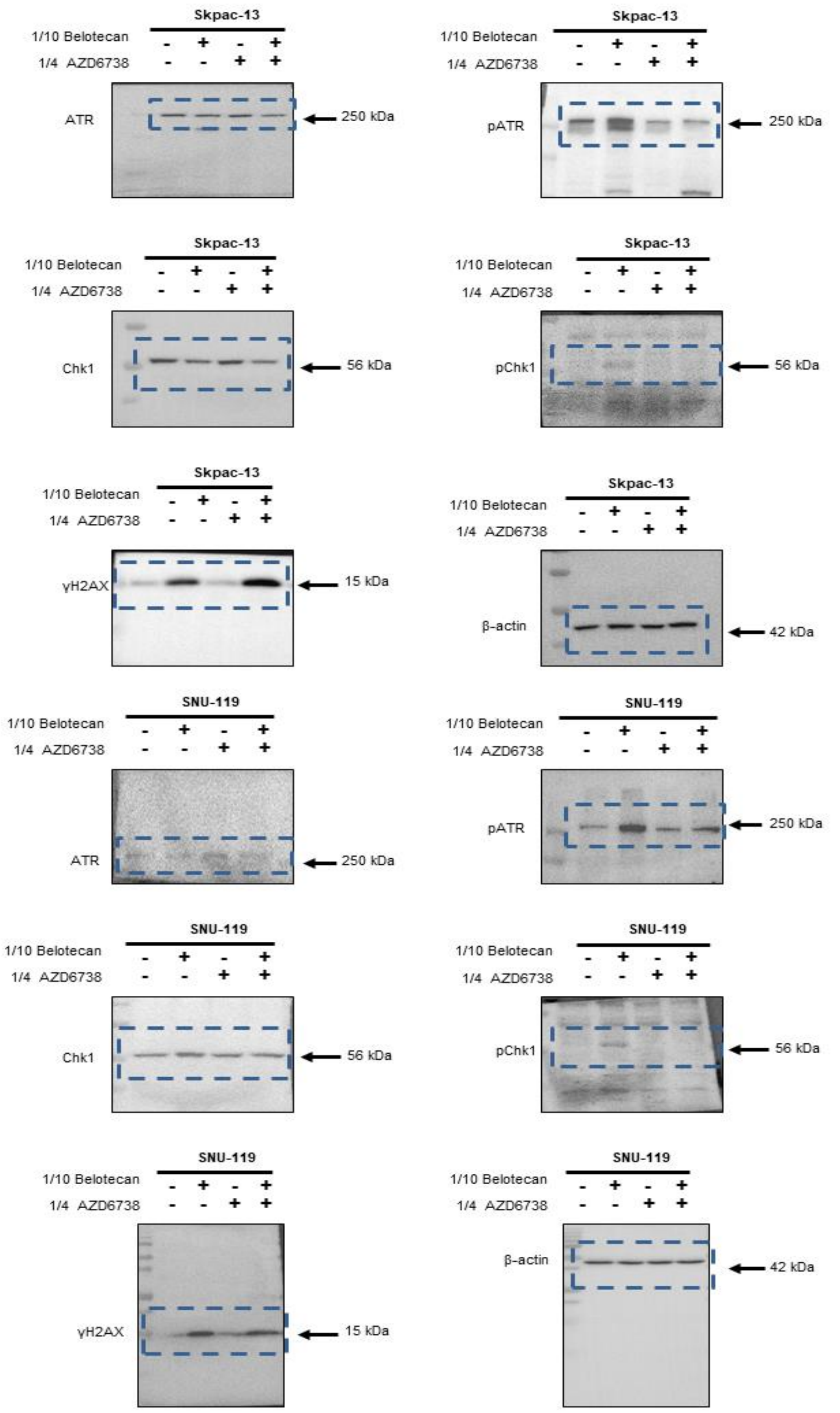
Skpac-13
1/10 Belotecan
1/4 AZD6738
ATR
250 kDa
pATR
Chk1
56 kDa
pChk1
γH2AX
15 kDa
β-actin
42 kDa
SNU-119

Raw western blot data of the cropped images shown in figure 3a. Western blotting of ATR, phospho-ATR, Chk1, phospho-Chk1 and γH2AX from Skpac-13 cells and SNU-119 cells. Each cells were treated with belotecan, AZD6738 and combination of both for (a) 24h and (b) 48h. β-actin was used as loading control.

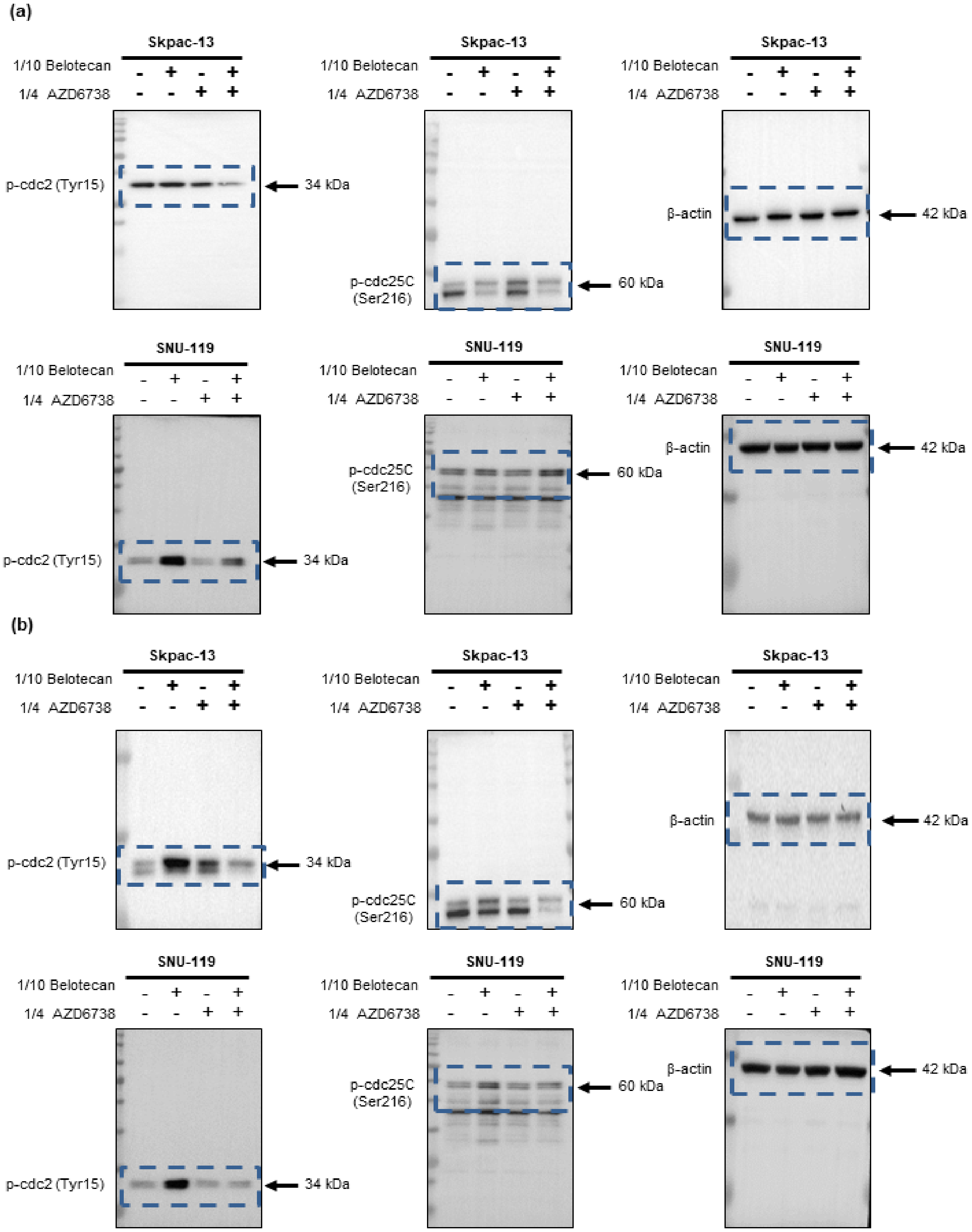

Raw western blot data of the cropped images shown in figure 4b. Western blotting of phospho-cdc2 (Tyr15) and phospho-cdc25C (Ser216) from Skpac-13 cells and SNU-119 cells. Each cells were treated with belotecan, AZD6738 and combination of both for (a) 24h and (b) 48h. β-actin was used as loading control.

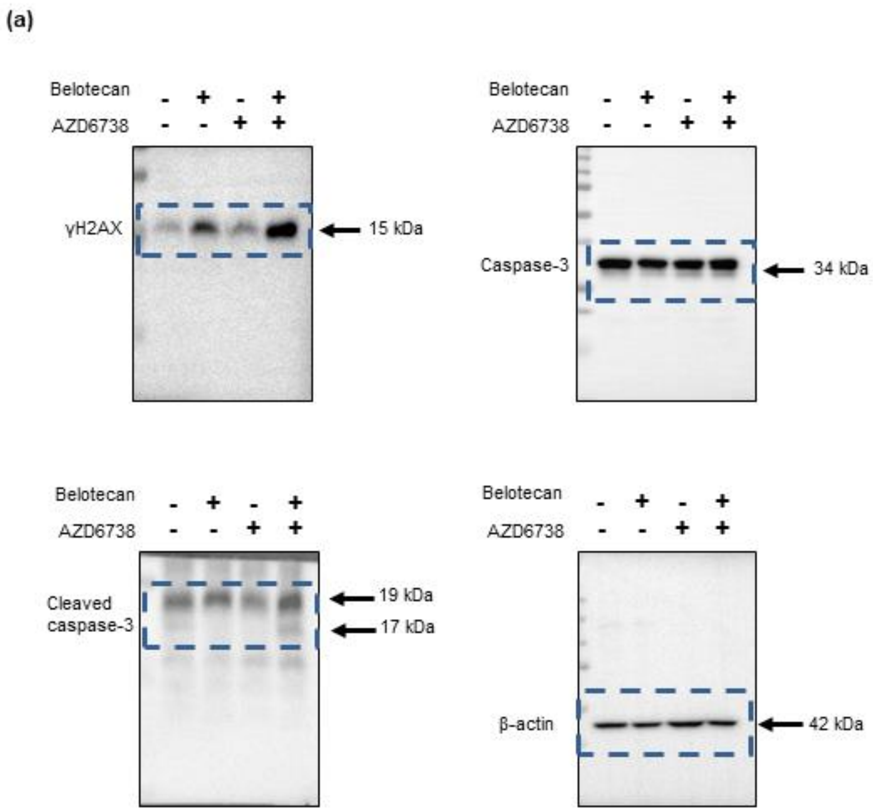

Raw western blot data of the cropped images shown in figure 5c. Western blotting of Caspase-3, Cleaved Caspase-3 and γH2AX from SKpac-13 xenograft tumor tissue. Each groups were injected with belotecan, AZD6738 and combination of both. β-actin was used as loading control.

© 2020 by the authors. Submitted for possible open access publication under the terms and conditions of the Creative Commons Attribution (CC BY) license (http://creativecommons.org/licenses/by/4.0/).
