# Supplementary material for: Synergism of AZD6738, an ATR Inhibitor, in Combination with Belotecan, a Camptothecin Analogue, in Chemotherapy-Resistant Ovarian Cancer
Source: Int J Mol Sci. 2021 Jan 27;22(3):1223. doi: 10.3390/ijms22031223 (PMC7865398; doi:10.3390/ijms22031223)
Supplement: Supplementary file 1 [file ijms-22-01223-s001.zip › ijms-1049612-supp-final-layout/supplementary figure and table.pdf]

International Journal of *Molecular Sciences*

MDPI

Article

Synergism of AZD6738, ATR inhibitor in combination with belotecan, Camptothecin analogue, in chemotherapy-resistant ovarian cancer

Jin Hur[1,2], Mithun Ghosh[1,2], Tae Heon Kim[3], Nahee Park[1], Kamal Pandey[1], Young Bin Cho[1], Sa Deok Hong[1,2], Nar Bahadur Katuwal[1,2], Minsil Kang[1], Hee Jung An[3], Yong Wha Moon[1*]

[1]Hematology and Oncology, Department of Internal Medicine, CHA Bundang Medical Center, CHA University, Seongnam, Korea; hurjinz@naver.com (J.H.); mithunghoshmg@gmail.com (M.G.); Skgml0413@naver.com (N.P.); pkamal@chauniv.ac.kr (K.P.); mypeacemaker@hanmail.net (Y.B.C.); duggy126@gmail.com (S.D.H.); narbahadurkatwal@gmail.com (N.B.K.); rkdalstlf1097@gmail.com (M.K.)

[2]Department of Biomedical Science, The Graduate School, CHA University, Seongnam, Korea; Ice_t69@cha.ac.kr (T.H.K.); hjahn@cha.ac.kr (H.-J.A.)

[3]Department of Pathology, CHA Bundang Medical Center, CHA University, Seongnam, Korea

*Correspondence: ymoon@cha.ac.kr (Y.W.M.); Tel: +82-31-780-3436; Fax: +82-31-780-3929

Supplementary figures:

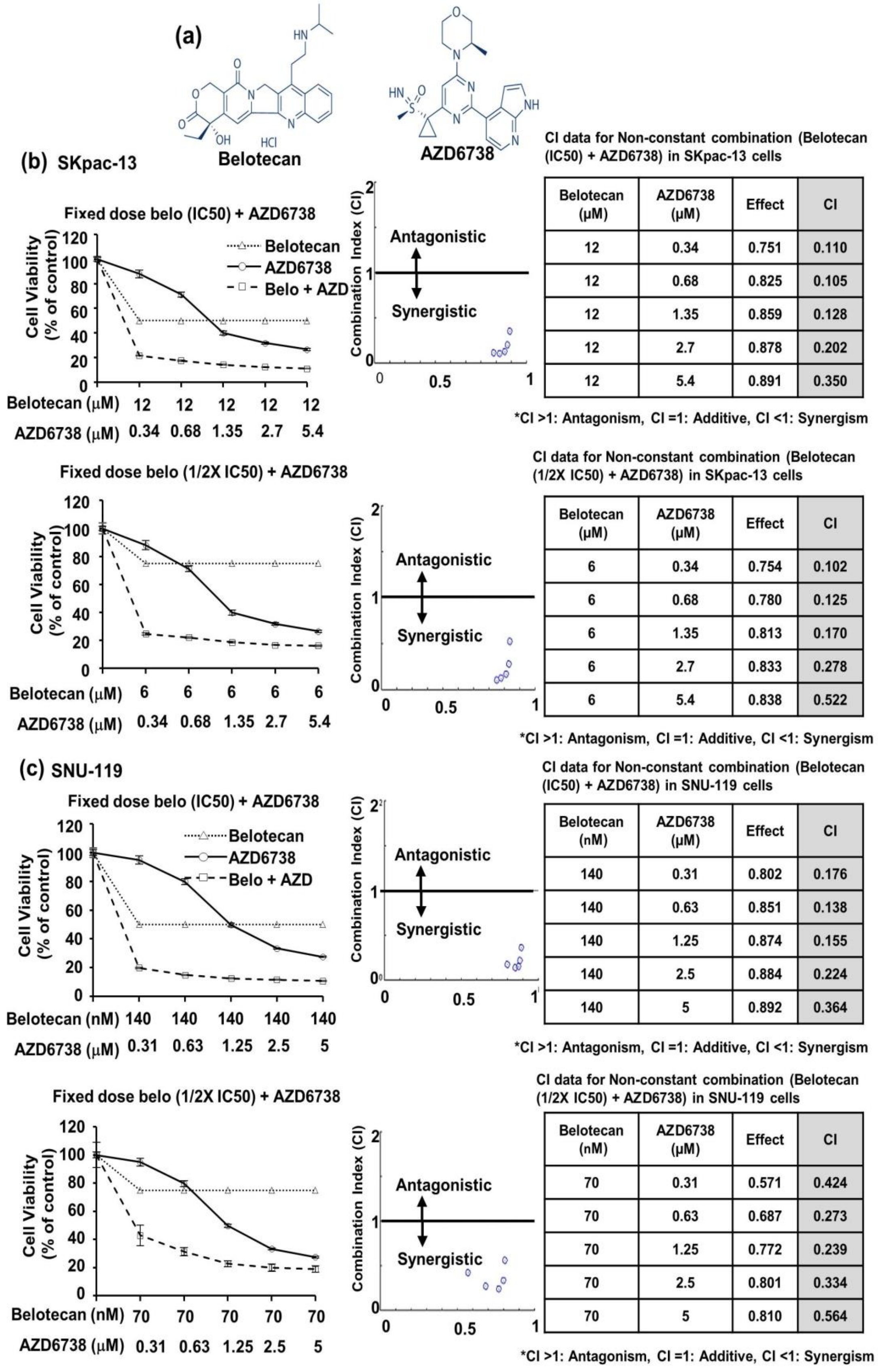

CI data for Non-constant combination (Belotecan (IC50) + AZD6738) in SKpac-13 cells

Belotecan (μM)	AZD6738 (μM)	Effect	CI
12	0.34	0.751	0.110
12	0.68	0.825	0.105
12	1.35	0.859	0.128
12	2.7	0.878	0.202
12	5.4	0.891	0.350

*CI >1: Antagonism, CI =1: Additive, CI <1: Synergism

CI data for Non-constant combination (Belotecan (1/2X IC50) + AZD6738) in SKpac-13 cells

Belotecan (μM)	AZD6738 (μM)	Effect	CI
6	0.34	0.754	0.102
6	0.68	0.780	0.125
6	1.35	0.813	0.170
6	2.7	0.833	0.278
6	5.4	0.838	0.522

*CI >1: Antagonism, CI =1: Additive, CI <1: Synergism

CI data for Non-constant combination (Belotecan (IC50) + AZD6738) in SNU-119 cells

Belotecan (nM)	AZD6738 (μM)	Effect	CI
140	0.31	0.802	0.176
140	0.63	0.851	0.138
140	1.25	0.874	0.155
140	2.5	0.884	0.224
140	5	0.892	0.364

*CI >1: Antagonism, CI =1: Additive, CI <1: Synergism

CI data for Non-constant combination (Belotecan (1/2X IC50) + AZD6738) in SNU-119 cells

Belotecan (nM)	AZD6738 (μM)	Effect	CI
70	0.31	0.571	0.424
70	0.63	0.687	0.273
70	1.25	0.772	0.239
70	2.5	0.801	0.334
70	5	0.810	0.564

*CI >1: Antagonism, CI =1: Additive, CI <1: Synergism

Figure S1. (**a**) Chemical structure of belotecan and AZD6738. (**b,c**) MTT assay indicated that various concentration of AZD6738, in combination with IC_{50} and half IC_{50} of belotecan synergize *in* Skpac-13 (**b**) and SNU-119 (**c**) epithelial ovarian cancer cell lines.

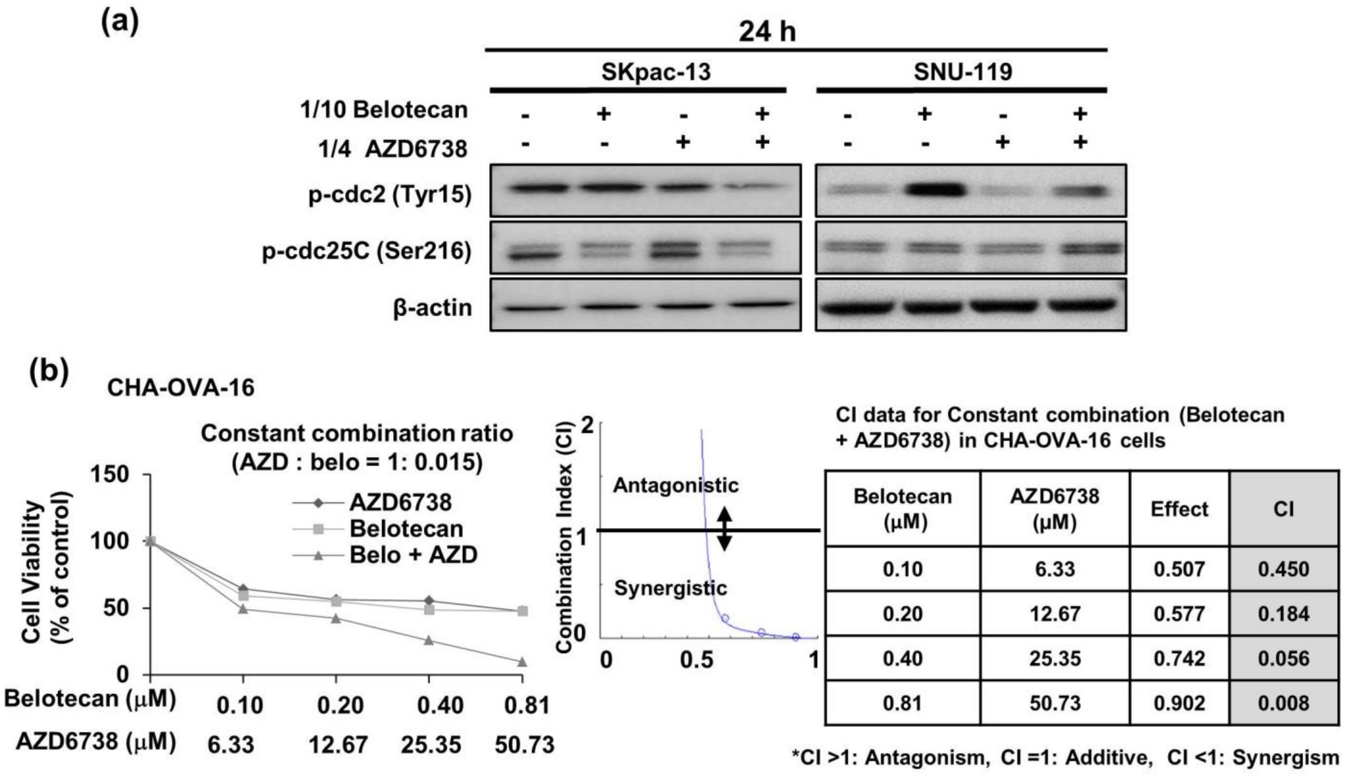

Belotecan (μM)	AZD6738 (μM)	Effect	CI
0.10	6.33	0.507	0.450
0.20	12.67	0.577	0.184
0.40	25.35	0.742	0.056
0.81	50.73	0.902	0.008

Figure S2. (a) Downstream genes of Chk1, cdc25c, and phospho-CDK1 were increasingly phosphorylated with belotecan at 24h. The expression of the proteins was determined by the western blot analysis and beta actin was used as loading control. (**b**) MTT assay demonstrated that Combination of belotecan and AZD6738—in a constant combination ratio of both drugs—demonstrated synergistic cytotoxicity in both CHA-OVA-16 primary ovarian cancer cells.

Supplementary tables:

Table S1. Various ovary cancer cell lines were screened for cytotoxicity of belotecan or AZD6738 alone.

Cell line	Origin	Chemosensitivity	Reference	Belotecan IC_{50}	AZD6738 IC_{50}
SKpac-13	Human	Paclitaxel-resistant	Notch3-specific Inhibition Using siRNA Knockdown or GSI Sensitizes Paclitaxel-Resistant Ovarian Cancer Cells	12 μM	2.7μM
A2780cis	Human	Cisplatin-resistant	Cancer Res 1987;47:414; Cancer Res 1988;48:5713	30nM	2.5μM
SNU-119	Human	Cisplatin-unknown<br>Paclitaxel - unknown		140nM	2.5μM
OVCAR-3	Human	Cisplatin-unknown<br>Paclitaxel-unknown		2nM	900nM
SKOV-3	Human	Cisplatin-unknown<br>Paclitaxel-unknown		19nM	1.7μM
ID8	Murine	Cisplatin-unknown<br>Paclitaxel-unknown		27nM	1.1μM

Table S2. Published and ongoing clinical trials of combined ATR inhibitor and topoisomerase inhibitor.

Published or ongoing clinical trial of combined ATRi and topoisomerase I inhibitor					
**Author**	**Journal**	**Drug**		**phase**	**Disease**
**(NCT number)**	**Year**	**ATR inhibitor**	**Topo I inhibitor**		
Thomas A NCT02487095	J ClinOncol 2018	VX-970	Topotecan	1,2	Small Cell Lung Cancer Ovarian Cancer Retinoblastoma Neuroendocrine Tumor Neuroepithelioma
NCT03896503		VX-970	Topotecan	2	Lung Cancer
NCT03641313		VX-970	Irinotecan	2	Gastric Cancer Gastroesophageal Cancer
NCT02595931		VX-970	Irinotecan Irinotecan Hydrochloride	1	Malignant Solid Neoplasm

Table S3. Primary antibodies used for western blot or Immunocytochemistry.

Antibody	Host species	Dilution	Company
ATR (Thr1989)	Mouse	1:1000	Santa Cruz, Dallas, Texas, USA
Phospho-ATR	Rabbit	1:1000	Gene Tex, CA 92606, USA
Chk1	Mouse	1:1000	Cell Signalling, Danvers, Massachusetts, USA
Phospho-Chk1(Ser345)	Rabbit	1:1000	Cell Signalling, Danvers, Massachusetts, USA
Caspase-3	Rabbit	1:1000	Cell Signalling, Danvers, Massachusetts, USA
Phospho-cdc25C (Ser216)	Rabbit	1:1000	Cell Signalling, Danvers, Massachusetts, USA
Phospho-cdc2 (Tyr15)	Rabbit	1:1000	Cell Signalling, Danvers, Massachusetts, USA
Phospho histone H2AX (Ser139/Tyr142)	Rabbit	1:1000	Cell Signalling, Danvers, Massachusetts, USA
Beta actin	Mouse	1:5000	Santa Cruz, Dallas, Texas, USA
Anti-Rabbit HRP	Goat	1:5000	Millipore, Burlington, Massachusetts, USA
Anti-Mouse HRP	Goat	1:5000	GeneTex, CA 92606, USA

© 2020 by the authors. Submitted for possible open access publication under the terms and conditions of the Creative Commons Attribution (CC BY) license (http://creativecommons.org/licenses/by/4.0/).
