# Supplementary figures and images for: Synergism of AZD6738, an ATR Inhibitor, in Combination with Belotecan, a Camptothecin Analogue, in Chemotherapy-Resistant Ovarian Cancer
Source: Int J Mol Sci. 2021 Jan 27;22(3):1223. doi: 10.3390/ijms22031223 (PMC7865398; doi:10.3390/ijms22031223)

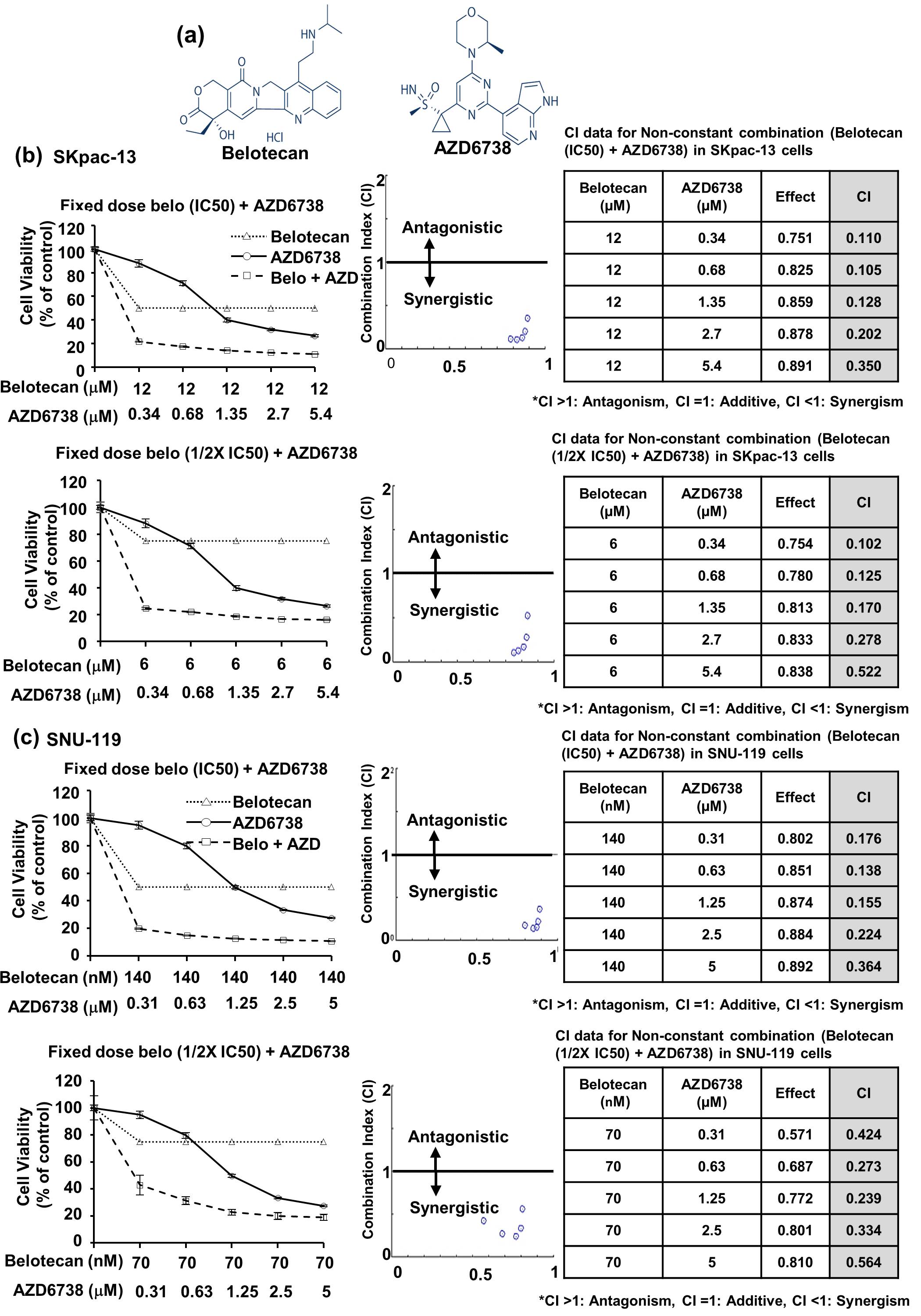

Supplement: Supplementary file 1 [file ijms-22-01223-s001.zip › ijms-1049612-supp-final-layout/Supplementary Fig1.jpg]

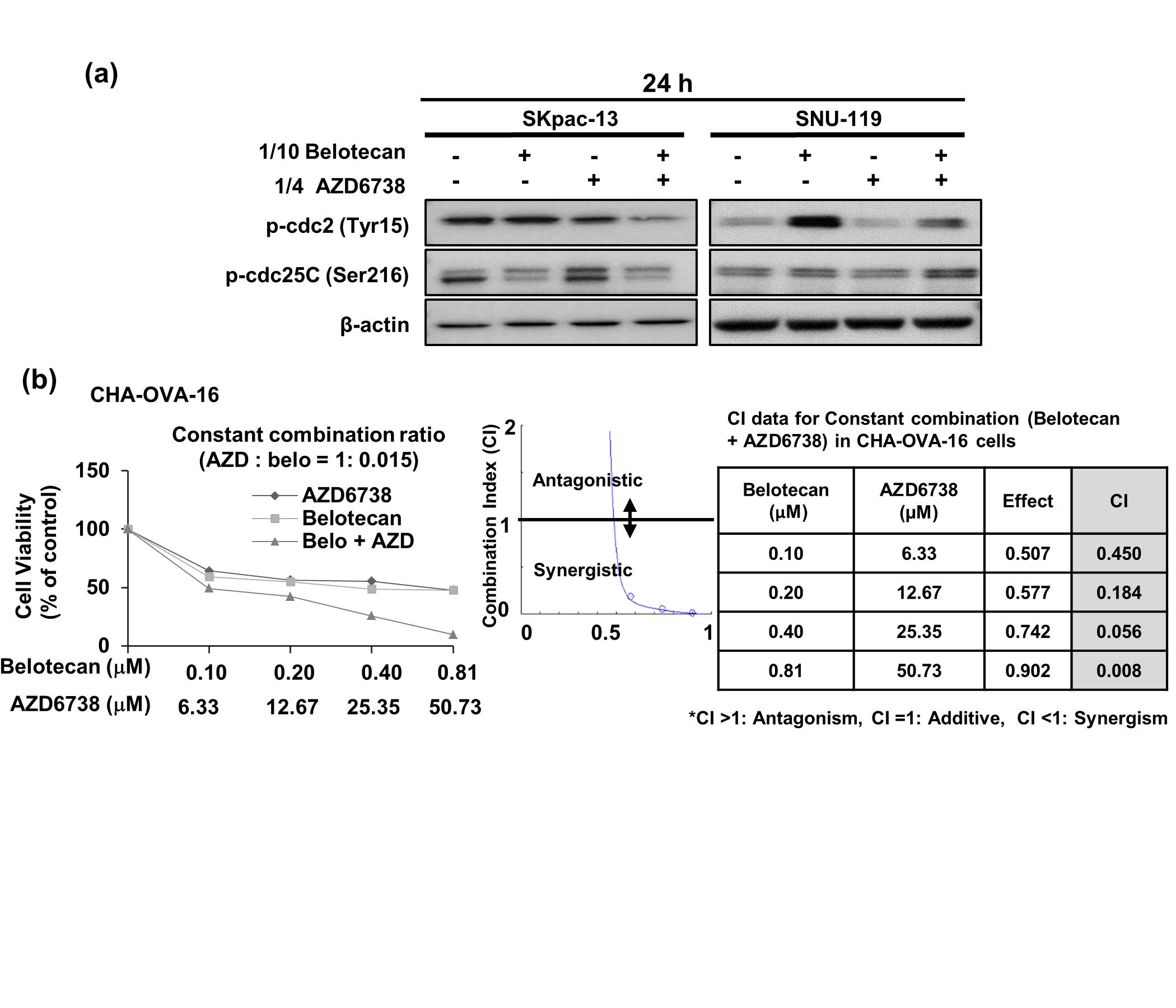

Supplement: Supplementary file 1 [file ijms-22-01223-s001.zip › ijms-1049612-supp-final-layout/Supplementary Fig2.jpg]
